# The effect of pubertal timing, as reflected by height tempo, on proximal femur shape: Findings from a population-based study in adolescents

**DOI:** 10.1016/j.bone.2019.115179

**Published:** 2020-02

**Authors:** Monika Frysz, Jennifer S. Gregory, Richard M. Aspden, Lavinia Paternoster, Jonathan H. Tobias

**Affiliations:** a Musculoskeletal Research Unit, Translational Health Sciences, Bristol Medical School, University of Bristol, Bristol, UK; b Aberdeen Centre for Arthritis and Musculoskeletal Health, University of Aberdeen, Aberdeen, UK; c Medical Research Council Integrative Epidemiology Unit, Bristol Medical School, University of Bristol, Bristol, UK; d Population Health Sciences, Bristol Medical School, University of Bristol, Bristol, UK

**Keywords:** ALSPAC, Proximal femur shape, Joint shape, Statistical shape modelling, Pubertal growth

## Abstract

**Objective:**

To examine the relationship between pubertal timing (using measures of height tempo) and proximal femur shape in a large adolescent cohort.

**Methods:**

Hip DXA scans were obtained in offspring from the Avon Longitudinal Study of Parents and Children. To quantify hip morphology, the images were analyzed using Shape software based on a 53-point statistical shape model and independent modes of variation (hip shape mode (HSM) scores) for each image were generated. Height tempo (which corresponds to age at peak height velocity (aPHV)) was estimated from serial height measurements collected between age 5–20 years. Multivariable linear regression was used to examine cross-sectional associations between height tempo and the top ten HSMs at age 14 and 18, adjusting for sex and fat mass index (FMI).

**Results:**

Complete outcome and covariate data were available from 3827 and 3507 participants at age 14 and 18 years, respectively. Mean aPHV was 13.5 and 11.8 years for males and females, respectively. At age 14, height tempo was associated with a majority of modes, except for HSM4 and there was strong evidence of interaction by sex. In males, all modes showed evidence of an association with tempo, independent of FMI, with the strongest observed for HSM8 (adjusted β 0.38 (0.33, 0.43) *p* = 4.1 × 10^−50^). Compared with males, the associations were generally weaker in females, with the strongest effect observed for HSM8 (adjusted β 0.10 (0.05, 0.14) *p* = 1.6 × 10^−5^). The overall effect of later pubertal timing on proximal femur shape in males was a narrower femoral neck and larger superolateral head, whereas in females these changes were hard to discern. When assessed at age 18, there was little relationship between tempo and proximal femur shape in either sex.

**Conclusion:**

Our results indicate that significant changes in hip shape occur during puberty, including aspects of shape which may be related to future risk of hip OA and/or fracture. However, puberty timing per se does not appear to exert long lasting effects on proximal femur shape.

## Highlights

• Puberty represents an important period for the development of hip shape.
• Associations between pubertal timing and hip shape at age 14 were stronger in males.
• Pubertal timing does not appear to have lasting effects on hip shape.

## 1 Introduction

Puberty is a period of rapid growth and is thought to be a critical period for bone development [1,2]. Furthermore, timing of puberty has been linked to a number of important health outcomes later in life [3]. For example, in the UK Biobank study, early menarche was associated with an increased risk of osteoarthritis (OA) and reduced risk of osteoporosis (OP) [3]. On the other hand, in the MRC NSHD cohort, later puberty was associated with lower aBMD at the hip and spine in men and women, which in turn affects the risk of fracture [2].

OP and OA are both common age-related conditions associated with ill health, morbidity and significant economic burden [4]. Despite identifying several risk factors, the exact mechanisms contributing to the risk of OA and OP in later life are not fully understood. Previous studies suggest that the shape of proximal femur is one of the most important risk factors for the development of hip OA [5,6]. In addition, hip shape has been found to be associated with hip fracture [[7], [8], [9]].

Previous studies of hip morphology have traditionally relied on measures of single geometrical indices, such as femoral neck width (FNW), neck-shaft angle or hip axis length [[10], [11], [12]]. However, these measurements are often highly correlated with each other and with measures of body size [13]. In contrast, statistical shape modelling (SSM), which uses landmark points to outline the contour of the proximal femur, provides a more comprehensive evaluation of femoral morphology. SSM has been previously successfully applied to study relationships of hip shape with both OA [14] and hip fracture [15].

While previous studies suggest that sex differences in bone geometry emerge around the time of puberty [16], it is unclear whether other aspects of hip structure, such as hip shape, are also related to puberty. This question has potential clinical relevance since, to the extent that significant changes in joint development occur during puberty, adverse factors around this time, like excessive mechanical loading, could theoretically exert long lasting influences on hip shape.

In the present study, we aimed to examine the relationship between pubertal timing (based on measures of height tempo) and proximal femur shape in the Avon Longitudinal Study of Parents and Children (ALSPAC) cohort. Whether puberty represents an important time for proximal femur shape development was investigated by examining relationships between height tempo and proximal femur shape at age 14. In addition, to determine whether puberty timing per se has long lasting effects on proximal femur shape, we investigated whether height tempo is related to proximal femur shape at age 18.

## 2 Methods

### 2.1 Study population

Pregnant women resident in Avon, UK with expected dates of delivery 1st April 1991 to 31st December 1992 were invited to take part in the study. The initial number of pregnancies enrolled was 14,541 (for these at least one questionnaire has been returned or a “Children in Focus” clinic had been attended by 19/07/99). Of these initial pregnancies, there was a total of 14,676 foetuses, resulting in 14,062 live births and 13,988 children who were alive at 1 year of age [17,18]. When the oldest children were approximately 7 years of age, an attempt was made to bolster the initial sample with eligible cases who had failed to join the study originally. As a result, when considering variables collected from the age of seven onwards (and potentially abstracted from obstetric notes) there are data available for more than the 14,541 pregnancies mentioned above. The total sample size for analyses using any data collected after the age of seven is therefore 15,247 pregnancies, resulting in 15,458 foetuses. Please note that the study website contains details of all the data that are available through a fully searchable data dictionary and variable search tool (http://www.bristol.ac.uk/alspac/researchers/our-data/). The outcome data and confounders in the present study were collected during Teen Focus (TF) 2 and TF 4 research clinics, whereas tempo was generated from height measurements collected at multiple time points (see below for more details). TF 2 was carried out between January 2005 and September 2006. Of 11,351 individuals invited 6147 attended. TF 4 clinic started in December 2008 and was completed by mid-2011. Of 10,101 individuals invited, 5217 attended.

Ethical approval for the study was obtained from the ALSPAC Ethics and Law Committee and the Local Research Ethics Committees.

### 2.2 Statistical shape modelling

To quantify the shape of proximal femur, SSM was applied to hip DXA images performed by GE Lunar Prodigy (Madison, WI, USA), collected at TF 2 and TF 4 clinics. Application of this method to ALSPAC data has been described previously [19]. Briefly, each image was analyzed in Shape software (University of Aberdeen, UK) to build a 53-point SSM. Landmark points were placed around each image to outline the shape of proximal femur including the acetabular eyebrow. This was followed by Procrustes analysis (to remove any translational, rotational and scaling information) and principal component analysis (PCA). In order to aid comparison between the time points, a pre-defined set of points previously obtained from an adult reference population [20] was applied to the adolescent data and independent modes of variation (hip shape modes (HSMs)), were subsequently generated. The first HSM accounts for the largest amount of variance in the dataset with subsequent modes accounting for less. Each individual is assigned a set of values for each HSM describing the distance from the mean shape in standard deviations (SDs). The first ten HSMs were used as outcomes in the analysis (please refer to Supplementary Table 1 for graphical representation and description of the variation described by the top ten HSMs based on the adult reference SSM).

### 2.3 Exposure and covariate data

Height was measured to the nearest 0.1 cm using a Harpenden stadiometer with shoes removed. These measurements were collected by trained field workers during assessment clinics, between age 5 and 20 years, and were used to estimate age at peak height velocity (aPHV) using SuperImposition by Translation and Rotation (SITAR) mixed effects growth curve analysis, a method previously described by Cole et al. [21]. This is a shape-invariant growth curve model consisting of a mean growth curve along with three transformations (size, tempo and velocity) to describe how each individual differs from the mean curve. Tempo (measured in years, derived for males and females separately) which corresponds to aPHV, reflects the timing of the growth process (negative values indicate early puberty and positive later puberty). A total of 20,849 height measurements for 2688 males, and 24,216 measurements for 3019 females were available for analysis. The derivation of aPHV in ALSPAC offspring has been described previously [22].

Previous studies suggest that fat mass or body mass index (BMI) affect the onset of puberty in males and females [23]. To adjust for the effect of fat mass, fat mass index (FMI) (independent of height) was calculated for each time point, as described previously [24]. Briefly, fat mass and height were log transformed. Log transformed fat mass was then regressed on log height, separately for males and females. Subsequently fat mass was regressed on height raised to the appropriate power (value corresponding to regression coefficient of log fat mass regressed on log height) and the residuals were used to adjust for FMI in analyses. Data on sex were obtained from hospital birth records.

### 2.4 Statistical analysis

The normality of data was explored using descriptive statistics and histograms. Descriptive statistics are expressed as means with SDs for continuous variables. Multivariable linear regression was used to examine cross-sectional associations between height tempo and the top ten HSMs at age 14 and 18, adjusting for sex (model 1) and additionally for FMI (model 2). Sex differences were explored by comparing regression coefficients and their 95% CIs in sex-stratified analysis and by formally testing for evidence of statistical interaction using likelihood-ratio tests. Additional sensitivity analyses, carried out in males and females separately, explored the differences in HSM scores at age 14 years, between individuals ≥90th and <90th sex-specific tempo percentiles and ≤10th and >10th sex-specific tempo percentiles, in unadjusted and FMI adjusted models. In order to aid comparison with regression results (when using tempo as a continuous measure), beta coefficients from sensitivity analyses were scaled to reflect one-year difference in tempo.

To illustrate the overall relationship between pubertal timing and proximal femur shape at each time point, sex-stratified coefficients from FMI adjusted linear regression models corresponding to 10th and 90th percentiles of tempo (each beta coefficient was multiplied by a value corresponding to 10th and 90th percentile of tempo to represent the differences between early vs. late maturers) were simultaneously entered into Shape software, for all modes showing evidence of an association with height tempo (*p*<0.005).

## 3 Results

### 3.1 Participant characteristics

Table 1 shows characteristics of study participants. Of individuals who attended TF 2 clinic (mean age at attendance 13.8 years), complete outcome and covariate data were available for 1797 males and 2030 females. Males who attended the assessment clinic were taller, had higher total body lean mass and lower total body fat mass compared with females. Of those who attended the TF 4 clinic (mean age at attendance 17.8 years), a total of 1597 males and 1910 females had complete outcome and covariate data. Similarly, to TF 2 results, males were taller, had higher lean mass content and lower fat mass content compared with females. Mean (SD) aPHV was 13.5 (0.9) and 11.8 (0.8) years for males and females, respectively. Mean values of tempo for males in 10th and 90th percentiles were − 1.41, 0.83 years and for females −1.41, 0.94 years, respectively, and corresponding mean values of aPHV for these percentiles were 12.1 and 14.4 years in males and 10.5 and 12.7 years in females (Supplementary Table 2).

**Table 1:** Descriptive statistics of ALSPAC study participants.

		Age 14 (TF 2)			Age 18 (TF 4)		
		N	Mean (SD)	p for sex diff^a^	N	Mean (SD)	p for sex diff^a^
Age at clinic attendance	M&F	3827	13.8 (0.2)	0.077	3507	17.7 (0.4)	0.612
	M	1797	13.8 (0.2)		1597	17.7 (0.4)	
	F	2030	13.8 (0.2)		1910	17.7 (0.4)	
Height (cm)	M&F	3827	163.6 (7.5)	<0.001	3507	171.6 (9.2)	<0.001
	M	1797	165.0 (8.5)		1597	178.8 (6.5)	
	F	2030	162.0 (6.2)		1910	165.5 (6.1)	
Weight (kg)	M&F	3827	54.6 (10.9)	0.675	3507	67.1 (13.4)	<0.001
	M	1797	54.8 (11.5)		1597	72.5 (13.3)	
	F	2030	54.5 (10.4)		1910	62.5 (11.7)	
Lean mass (kg)	M&F	3827	38.1 (6.4)	<0.001	3507	45.9 (9.9)	<0.001
	M	1797	41.2 (7.1)		1597	55.2 (6.1)	
	F	2030	35.3 (4.1)		1910	38.1 (4.0)	
Fat mass (kg)	M&F	3827	13.9 (8.0)	<0.001	3507	18.0 (10.3)	<0.001
	M	1797	11.1 (7.6)		1597	14.1 (10.1)	
	F	2030	16.3 (7.5)		1910	21.3 (9.2)	
Tempo (years)^b^	M&F	3827	0.03 (0.8)		3507	0.01 (0.8)	
	M	1797	0.02 (0.8)		1597	−0.02 (0.8)	
	F	2030	0.04 (0.9)		1910	0.03 (0.9)	
aPHV (years)	M&F	3827	12.6 (1.2)	<0.001	3507	12.6 (1.2)	<0.001
	M	1797	13.5 (0.9)		1597	13.5 (0.9)	
	F	2030	11.8 (0.8)		1910	11.8 (0.8)	

Abbreviations: M (males), F (females), aPHV (age at peak height velocity).

a Unpaired *t*-test to assess the null hypothesis of no difference in distributions between males and females at each time point.

b Tempo corresponds to the timing of pubertal growth spurt (and thus aPHV) in each individual compared with the sex-specific means. Geometrically it indicates subject-specific left-right shift or translation in the spline curve. Negative values indicate early puberty, and positive values indicate late puberty. Please note that tempo measure in this sample is not equal to 0 because tempo was generated on a larger sample of individuals compared with the sample used in current analyses.

### 3.2 Relationship between tempo and proximal femur shape at age 14

In sex-combined analysis (model 1), tempo was associated with the majority of HSMs, except for HSM4 (Table 2). Following adjustment for FMI (model 2) associations were essentially unchanged. There was strong evidence of interaction by sex (in both models) for all HSMs except HSM6 (Table 2).

**Table 2:** Associations between tempo and the top ten HSMs in ALSPAC adolescents at age 14 (*N* = 3827).

HSM	Model 1			Model 2		
	β (95% CI)	p	p for sex-int	β (95% CI)	p	p for sex-int
1	−0.04 (−0.06, −0.03)	1.4 × 10^−8^	5.3 × 10^−5^	−0.04 (−0.06, −0.03)	3.8 × 10^−8^	2.6 × 10^−5^
2	0.15 (0.12, 0.18)	4.5 × 10^−25^	6.8 × 10^−9^	0.13 (0.10, 0.15)	2.6 × 10^−18^	9.7 × 10^−15^
3	0.14 (0.12, 0.17)	9.2 × 10^−28^	2 × 10^−13^	0.12 (0.10, 0.15)	7.4 × 10^−22^	5.0 × 10^−19^
4	0.02 (−0.00, 0.05)	0.073	0.001	0.02 (−0.00, 0.05)	0.093	0.001
5	0.14 (0.11, 0.17)	1.1 × 10^−21^	1.9 × 10^−21^	0.15 (0.12, 0.18)	4.8 × 10^−24^	3.2 × 10^−19^
6	−0.04 (−0.07, −0.02)	0.001	0.758	−0.06 (−0.08, −0.03)	6.4 × 10^−6^	0.385
7	−0.06 (−0.08, −0.03)	2.4 × 10^−6^	1.6 × 10^−14^	−0.07 (−0.09, −0.04)	1.6 × 10^−8^	1.2 × 10^−11^
8	0.25 (0.21, 0.28)	7.8 × 10^−48^	8.7 × 10^−14^	0.23 (0.20, 0.27)	5.3 × 10^−43^	1.1 × 10^−16^
9	0.12 (0.09, 0.15)	7.2 × 10^−16^	8.6 × 10^−8^	0.12 (0.09, 0.15)	2.0 × 10^−16^	2.5 × 10^−7^
10	0.06 (0.04, 0.08)	6.4 × 10^−8^	0.038	0.05 (0.03, 0.07)	1.7 × 10^−5^	0.001

Abbreviations: HSM (hip shape mode), CI (confidence interval). Table shows results of linear regression analysis between tempo and the top ten HSMs in male and female adolescents. Regression coefficients represent SD change in HSM per one-year increase in height tempo, 95% CIs and *p* value. Model 1: adjusted for sex; model 2: model 1 + fat mass index.

In males, in unadjusted analysis there was evidence of association of tempo with all modes (model 1), and these results were essentially unchanged following FMI adjustment (model 2) (Table 3).

**Table 3:** Associations between tempo and the top ten HSMs in ALSPAC at age 14, stratified by sex.

	HSM	Model 1		Model 2	
		β (95% CI)	p	β (95% CI)	p
Males	1	−0.08 (−0.10, −0.06)	1.2 × 10^−10^	−0.08 (−0.10, −0.06)	1.1 × 10^−10^
	2	0.24 (0.20, 0.28)	2.8 × 10^−29^	0.24 (0.20, 0.28)	9.8 × 10^−31^
	3	0.24 (0.21, 0.28)	1.6 × 10^−36^	0.25 (0.21, 0.28)	9.2 × 10^−38^
	4	0.07 (0.03, 0.11)	4.2 × 10^−4^	0.07 (0.03, 0.11)	4.4 × 10^−4^
	5	0.30 (0.26, 0.34)	3.0 × 10^−41^	0.30 (0.26, 0.34)	3.4 × 10^−41^
	6	−0.05 (−0.09, −0.01)	0.02	−0.05 (−0.09, −0.01)	0.025
	7	−0.16 (−0.19, −0.12)	3.0 × 10^−19^	−0.15 (−0.19, −0.12)	4.3 × 10^−19^
	8	0.38 (0.33, 0.43)	1.6 × 10^−49^	0.38 (0.33, 0.43)	4.1 × 10^−50^
	9	0.20 (0.16, 0.24)	1.2 × 10^−21^	0.20 (0.16, 0.24)	1.2 × 10^−21^
	10	0.09 (0.05, 0.12)	4.4 × 10^−7^	0.09 (0.06, 0.12)	2.0 × 10^−7^
Females	1	−0.02 (−0.04, 0.00)	0.109	−0.01 (−0.03, 0.01)	0.196
	2	0.07 (0.04, 0.11)	1.2 × 10^−4^	0.01 (−0.02, 0.05)	0.476
	3	0.06 (0.02, 0.09)	0.001	0.01 (−0.02, 0.05)	0.498
	4	−0.01 (−0.05, 0.02)	0.379	−0.03 (−0.06, 0.01)	0.126
	5	0.02 (−0.02, 0.06)	0.416	0.04 (−0.00, 0.08)	0.052
	6	−0.04 (−0.07, −0.01)	0.012	−0.07 (−0.10, −0.03)	6.1 × 10^−5^
	7	0.03 (−0.01, 0.06)	0.109	0.01 (−0.02, 0.04)	0.498
	8	0.13 (0.09, 0.18)	1.3 × 10^−9^	0.10 (0.05, 0.14)	1.6 × 10^−5^
	9	0.05 (0.01, 0.09)	0.017	0.06 (0.02, 0.10)	0.006
	10	0.04 (0.01, 0.07)	0.007	0.02 (−0.01, 0.05)	0.303

Abbreviations: HSM (hip shape mode), CI (confidence interval). Table shows results of linear regression analysis between tempo and the top ten HSMs in male (*N* = 1797) and female (*N* = 2030) adolescents. Results are SD change in HSM per one-year increase in height tempo, 95% CIs and p value. Model 1: unadjusted; model 2: adjusted for fat mass index.

In females, associations between tempo and HSMs were generally weaker compared with males. There was evidence for an association with a number of modes, including HSM2, HSM3, HSM6, HSM8, HSM9 and HSM10 (model 1, Table 3). Following adjustment for FMI (model 2) associations with HSM2, HSM3 and HSM10 attenuated towards the null and there was no longer evidence of an association, the association with HSM6 was strengthened, whereas HSM8 and HSM9 results remained unchanged (Table 3).

**Fig. 1:**
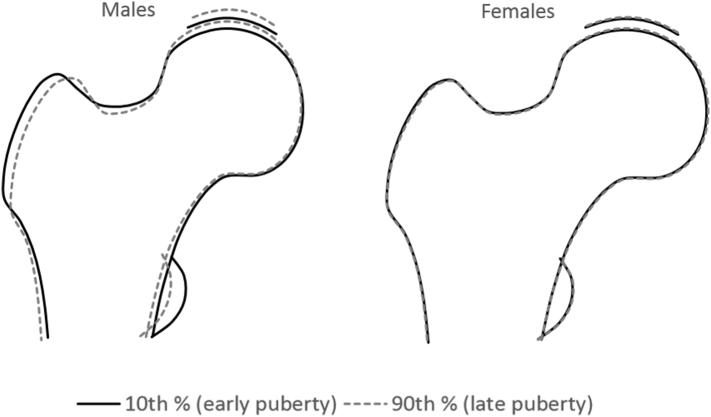
The overall difference in proximal femur shape at age 14 between early vs. late matures (changes in proximal femur shape associated with unit change in tempo) based on adjusted (model 2) beta coefficients. Beta coefficients were scaled to reflect changes in early maturers (10th percentile of tempo) vs. late maturers (90th percentile of tempo).

We modelled overall proximal femur shape in early versus late maturers, reflected by 10th and 90th tempo percentile respectively, adjusted for FMI (Fig. 1). In males, later puberty was characterised at age 14 by slight narrowing of FNW, smaller lesser and greater trochanters and larger femoral head in the superolateral aspect. In females, the overall relationship between pubertal timing and proximal femur shape at age 14 was harder to discern.

### 3.3 Sensitivity analyses

Compared with the main results when using tempo as a continuous measure, in males, the differences in HSM scores between late maturers vs. the rest (individuals in 90th percentile of tempo and above vs. those below 90th percentile of tempo), were largely consistent in terms of the direction and size of the effect (models 1 and 2) (Supplementary Table 3).

Analyses in late maturing females likewise revealed similar results to those of tempo as a continuous measure, with the exception that differences were now more pronounced in HSM5 scores (adjusted beta = 0.12 (0.03, 0.20) *p* = 3.2 × 10^−5^ vs. 0.04 (−0.00, 0.08) *p*=0.052 when using tempo as a continuous measure).

Supplementary Table 4 shows the differences in HSM scores between early maturers vs. the rest (individuals in 10th percentile of tempo vs. those above 10th percentile of tempo). Compared with the main results, in males, all coefficients were in the opposite direction (except for HSM4, which showed no evidence of differences in scores between early maturers vs. the rest) and broadly similar in terms of magnitude of effect (models 1 and 2), except HSM6 and HSM7 which were larger. In females, in unadjusted analysis there was some evidence for differences in HSM2 and HSM7 scores between early maturers vs. the rest, however these associations attenuated towards the null following adjustment for FMI.

### 3.4 Relationship between tempo and proximal femur shape at age 18

Compared with age 14 results, associations between tempo and proximal femur shape at age 18 were generally weaker, as reflected by considerably lower beta coefficients. In sex-combined analysis, based on model 1, there was evidence of association between tempo and HSM1, HSM2, HSM5, HSM9 and HSM10 (Table 4). Following adjustment for FMI (model 2), associations with HSM2 and HSM10 were fully and partly attenuated respectively, and there was now weak evidence for an association with HSM8.

**Table 4:** Associations between tempo and the top ten HSMs in ALSPAC adolescents at age 18 (*N* = 3507).

HSM	Model 1			Model 2		
	β (95% CI)	p	p for sex-int	β (95% CI)	p	p for sex-int
1	0.02 (0.00, 0.03)	0.031	0.756	0.02 (0.00, 0.04)	0.014	0.762
2	0.05 (0.02, 0.08)	0.003	0.186	0.00 (−0.03, 0.04)	0.789	0.197
3	0.00 (−0.02, 0.03)	0.823	0.178	−0.02 (−0.05, 0.00)	0.092	0.159
4	−0.00 (−0.03, 0.03)	0.889	0.172	−0.01 (−0.04, 0.02)	0.523	0.168
5	−0.06 (−0.09, −0.03)	2.6 × 10^−4^	0.149	−0.06 (−0.09, −0.03)	2.7 × 10^−4^	0.148
6	−0.01 (−0.04, 0.02)	0.488	0.670	−0.02 (−0.05, 0.01)	0.235	0.679
7	0.01 (−0.01, 0.04)	0.306	0.880	−0.01 (−0.04, 0.02)	0.490	0.847
8	−0.01 (−0.04, 0.03)	0.650	0.079	−0.04 (−0.08, −0.01)	0.021	0.068
9	−0.08 (−0.11, −0.05)	3.9 × 10^−6^	0.766	−0.07 (−0.11, −0.04)	2.4 × 10^−5^	0.761
10	0.07 (0.04, 0.10)	5.3 × 10^−6^	0.677	0.03 (0.00, 0.06)	0.038	0.627

Abbreviations: HSM (hip shape mode), CI (confidence interval). Table shows results of linear regression analysis between tempo and the top ten HSMs in male and female adolescents. Regression coefficients represent unit change in HSM per one-year increase in height tempo, 95% CIs and p value. Model 1: adjusted for sex; model 2: model 1 + fat mass index.

There was little evidence to suggest interaction by sex, however sex stratified analyses suggested that associations between tempo and HSM2 and HSM5 observed in sex combined analyses were largely driven by those in females (Table 5).

**Table 5:** Associations between tempo and the top ten HSMs in ALSPAC at age 18, stratified by sex.

Males	HSM	Model 1		Model 2	
		β (95% CI)	p	β (95% CI)	p
	1	0.02 (−0.01, 0.05)	0.119	0.02 (−0.00, 0.05)	0.08
	2	0.02 (−0.02, 0.07)	0.333	−0.01 (−0.06, 0.03)	0.613
	3	0.02 (−0.02, 0.06)	0.235	0.00 (−0.03, 0.04)	0.831
	4	0.02 (−0.02, 0.06)	0.342	0.02 (−0.02, 0.06)	0.352
	5	−0.03 (−0.08, 0.01)	0.163	−0.03 (−0.08, 0.01)	0.167
	6	−0.02 (−0.07, 0.03)	0.455	−0.03 (−0.08, 0.02)	0.303
	7	0.02 (−0.02, 0.05)	0.396	−0.00 (−0.04, 0.03)	0.841
	8	0.03 (−0.03, 0.08)	0.314	−0.01 (−0.06, 0.05)	0.77
	9	−0.09 (−0.13, −0.04)	2.4 × 10^−4^	−0.08 (−0.13, −0.03)	7.7 × 10^−4^
	10	0.08 (0.03, 0.12)	8.8 × 10^−4^	0.04 (−0.01, 0.08)	0.118
Females	1	0.02 (−0.00, 0.04)	0.131	0.02 (−0.00, 0.04)	0.087
	2	0.07 (0.02, 0.11)	0.002	0.01 (−0.03, 0.06)	0.513
	3	−0.01 (−0.05, 0.02)	0.482	−0.04 (−0.08, −0.01)	0.013
	4	−0.02 (−0.06, 0.02)	0.32	−0.03 (−0.07, 0.00)	0.08
	5	−0.08 (−0.12, −0.04)	4.2 × 10^−4^	−0.08 (−0.13, −0.04)	4.2 × 10^−4^
	6	−0.01 (−0.05, 0.04)	0.801	−0.01 (−0.06, 0.03)	0.501
	7	0.01 (−0.03, 0.05)	0.524	−0.01 (−0.05, 0.02)	0.45
	8	−0.04 (−0.08, 0.01)	0.128	−0.07 (−0.11, −0.02)	0.005
	9	−0.08 (−0.12, −0.03)	0.002	−0.07 (−0.12, −0.02)	0.006
	10	0.07 (0.02, 0.11)	0.002	0.03 (−0.01, 0.07)	0.144

Abbreviations: HSM (hip shape mode), CI (confidence interval). Table shows results of linear regression analysis between tempo and the top ten HSMs in male (N = *N* = 1597) and female (*N* = 1910) adolescents. Results are SD change in HSM per one-year increase in height tempo, 95% CIs and p value. Model 1: unadjusted; model 2: adjusted for fat mass index.

**Fig. 2:**
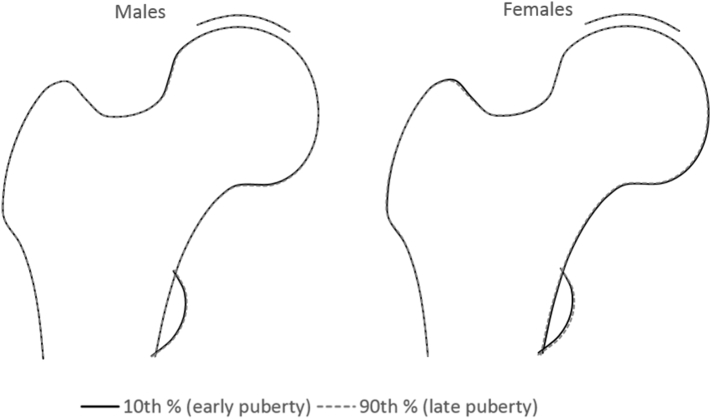
The overall difference in proximal femur shape at age 18 between early vs. late matures (changes in proximal femur shape associated with unit change in tempo) based on adjusted (model 2) beta coefficients. Beta coefficients were scaled to reflect changes in early maturers (10th percentile of tempo) vs. late maturers (90th percentile of tempo).

When modelling overall proximal femur shape at age 18 according to pubertal timing, the differences between early vs. late maturers were hard to discern in both sexes (Fig. 2).

## 4 Discussion

We examined the association between pubertal timing (as measured by height tempo) and proximal femur shape in ALSPAC adolescents. In males, tempo was strongly associated with many of the top 10 HSMs derived from hip DXA scans performed at age 14, with little attenuation after adjustment for FMI. In contrast, much weaker associations were observed between tempo and proximal femur shape at this age in females. At age 18, associations between pubertal timing and proximal femur shape were considerably weaker compared with age 14 results.

In the case of male participants, age of first clinic attendance closely corresponded to the mean aPHV (13.8 and 13.5 years, respectively). Therefore, in our analyses in males, we were able to examine how proximal femur shape varies across the full spectrum of pubertal development, enabling us to infer how proximal femur shape alters during puberty, despite our cross-sectional study design. Having constructed an overall effect on proximal femur shape by combining associations with all HSMs related to tempo, our results infer that as puberty advances, FNW increases, both the lesser and greater trochanters enlarge, and there is some re-shaping of the superolateral aspect of the femoral head. In contrast, in female participants, age of first clinic attendance was two years beyond mean aPHV (13.8 and 11.8 years, respectively). Therefore, our analyses in females only provided limited information as to how proximal femur shape alters during puberty, presumably explaining the considerably weaker association between tempo and proximal femur shape at age 14 in girls compared to boys. However, the availability of HSM data from post-pubertal males and females allowed us to investigate if there are any lasting effects of pubertal timing on proximal femur shape in both sexes.

The relationship between FNW and variation in femoral head have been previously investigated in relation to both osteoporotic fracture and hip OA. For example, Castano-Betancourt et al. reported an association between wider FNW and higher risk of incident hip OA [25]. In terms of FNW-hip fracture associations, the evidence is conflicting with previous studies reporting associations between both, wider and narrower FNW and hip fracture [8,11,26,27]. Finally, a previous study investigating radiographic patterns of OA in older men and women (mean age 66 years) found clear associations of male sex with superolateral OA while females showed a tendency to more medial patterns [28].

To our knowledge, this is the first study to investigate cross-sectional relationships between pubertal stage and proximal femur shape derived from SSM applied to hip DXA scans obtained concurrently. Our findings are consistent with a previous study by Pujol et al. in boys aged between 9 and 16 years, who found that the size of femoral head and greater and lesser trochanters increased with age [29]. The present findings are also consistent with our previous study based on the same cohort, where we observed a positive relationship between Tanner stage and FNW derived from hip structural analysis applied to the same hip DXA scans [16]. However, in contrast to the present results, we previously found that if anything, Tanner stage had a stronger association with FNW in females compared with males. One possible explanation for this apparent disparity is that in the latter study, due to the nature of Tanner staging, late maturing girls were in a separate category, and had a relatively strong influence on the results despite their small number. That said, in the present study where we also examined proximal femur shape in a separate category of late maturing girls, defined as >90th tempo centile, broadly similar relationships were observed between tempo and proximal femur shape to those seen in analyses based on tempo as a continuous measure.

Pubertal growth might represent a sensitive period for the development of an unfavourable bone phenotype leading to increased risk of fracture or OA in later life. As suggested by Bass et al., during periods of fast growth (as in puberty) specific bone regions might be more responsive to genetic and/or environmental stimuli, compared with periods of steady growth or with stages near or at completion of growth [30]. Consistent with this suggestion, Siebenrock et al. showed that cam-type deformity arises in childhood, often as a result of high impact sporting activity [31]. In addition, a previous study in pre-professional soccer players (mean age 14.4), followed up for a mean period of 2.4 years, noted increases in cam-type deformities between age 12 and 14 years, which continued to increase from the age of 14 until closure of the growth plate [32]. Furthermore, Packer et al. suggested that high impact activities and injuries taking place around critical periods of development can result in cam-type deformities, whereas cam-deformity prevalence and severity remained unchanged following growth plate closure [33]. Further studies are justified to examine whether changes in hip shape during puberty which we found predispose to deformities occurring around this time. Although cam-type deformities are difficult to detect by SSM as currently employed, it may be possible to overcome this limitation by the development of regional hip shape models [34].

Previous research has found relationships between pubertal timing and adverse musculoskeletal outcomes in later life. For example, a study based on UK Biobank reported associations between early menarche and increased risk of OA as well as between later menarche and increased risk of OP [3]. Similarly, a previous study by Finkelstein et al. found that men with a history of delayed puberty had lower femoral neck BMD compared with men with normal pubertal timing [35]. Several of the HSMs found to be associated with pubertal timing in our study, namely HSM2, HSM3, HSM4 and HSM9, were also found to be associated with radiographic hip OA in the MrOS cohort, which used the same SSM template used here (B Faber et al., submitted for publication). Although age at onset of walking in infancy has been shown to be associated with variations in hip shape in older age [36] in this study we found little relationship between height tempo and proximal femur shape measured at age 18, suggesting that pubertal timing per se does not influence hip shape in later life.

### 4.1 Strengths and limitations

To our knowledge, this study represents the first application of SSM to describe variation in proximal femur shape in adolescents. Another strength is our use of growth tempo as a measure of pubertal timing, given its close relationship with skeletal maturation. A further advantage of growth tempo is that this relies on objective measurements, in contrast to Tanner stage, which was determined in ALSPAC by self-completed questionnaires. Use of PHV as a measure of puberty is supported by previous good agreement between PHV and secondary sexual characteristics, such as testicular volume [37] and age at menarche [38]. In our study, the correlation between Tanner stage categories (based on pubic hair development) and aPHV was −0.63 and −0.64 in males and females, respectively (those in later Tanner stage categories reached PHV at an earlier age). Supplementary Fig. 1 shows a scatterplot of aPHV vs. Tanner stage category. A further strength was the incorporation of Procustes analysis in the derivation of hip shape, which removes scaling differences between images, thereby ensuring that relationships between puberty and hip shape which we observed were not simply a reflection of changes in overall skeletal size. In terms of limitations, whereas timing of the age 14 DXA assessment was well suited to studying cross-sectional relationships between tempo and proximal femur shape in males, this was less than ideal in females, of whom the great majority were well past PHV at this age. In order to address this issue, additional sensitivity analyses compared late maturing females vs. the rest, however the results were consistent with those when using tempo as a continuous measure. As previously highlighted [39], cross-comparison of individual HSMs between studies is difficult, since each SSM is unique to data it is derived from. However, a pre-defined set of points was applied in this study, which overcomes this limitation and allows direct comparison of hip shape between time points and with other studies using the same SSM template. While this is the first time a pre-defined set of points from an adult population has been applied to adolescent data, we were able to show that the model still produced 10 independent modes of variation [19]. At least theoretically, one of the limitations in applying a pre-specified model obtained from a different population is that, if there are significant differences in hip shape compared with the reference population, the pre-specified model may not be optimised for describing hip shape, and hence variability in hip shape may not be completely captured. Finally, as with any cross-sectional study, some caution needs to be exercised in attributing causal relationships. That said, other than fat mass index (measure of fat mass independent of height) which we adjusted for, it is difficult to conceive of other confounding factors which might have contributed to the relationship we observed between tempo and proximal femur shape.

## 5 Conclusions

We investigated relationships between pubertal timing (as reflected by height tempo) and proximal femur shape in a large population-based cohort of adolescents. At age 14, we found strong cross-sectional associations between tempo and multiple HSMs in males, together suggesting that as puberty advances, FNW increases, both the lesser and greater trochanters enlarge, and there is some re-shaping of the superolateral aspect of the femoral head. Whereas these relationships were much weaker in females, this is likely to reflect the fact that the great majority had already reached PHV by this time. Taken together, our results indicate that significant changes in hip shape occur during puberty, including aspects of shape which may be related to future risk of hip OA and/or fracture. On the other hand, little relationship was observed between tempo and proximal femur shape derived from DXA scans performed at age 18, in either sex, suggesting that age of puberty does not exert lasting effect on proximal femur shape.

## Author contributions

Conceptualization JT, LP, MF; Data curation MF, JG, RA; Formal analysis MF; Funding acquisition MF, LP, JT; Investigation MF; Methodology JG, RA, MF; Software JG, RA; Supervision JT, LP; Writing - original draft MF; Writing - review & editing MF, JG, RA, LP, JT.

## Funding

The UK Medical Research Council and the Wellcome Trust (ref: 102215/2/13/2) and the University of Bristol provide core support for ALSPAC. MF was supported by a Wellcome Trust PhD studentship (ref: 105504/Z/14/Z). LP works in the Medical Research Council Integrative Epidemiology Unit at the University of Bristol which is supported by the Medical Research Council and the University of Bristol (MC_UU_00011/1). A comprehensive list of grants funding is available on the ALSPAC website (http://www.bristol.ac.uk/alspac/external/documents/grant-acknowledgements.pdf). Age 17 clinical assessment was specifically funded by Wellcome Trust (084632/Z/08/Z).

This publication is the work of the authors and MF will serve as guarantor for the contents of this paper. None of the funders had any influence on data collection, analysis, interpretation of the results, or writing of the paper.

## Declaration of competing interest

All authors have no conflicts of interest to declare.

## Appendix A Supplementary data

Supplementary material Image 1
