# Supplementary material for: The effect of pubertal timing, as reflected by height tempo, on proximal femur shape: Findings from a population-based study in adolescents
Source: Bone. 2020 Feb;131:115179. doi: 10.1016/j.bone.2019.115179 (PMC6961111; doi:10.1016/j.bone.2019.115179)
Supplement: Supplementary file 1 — Supplementary material [file mmc1.docx]

**The effect of pubertal timing, as reflected by height tempo, on proximal femur shape: findings from a population-based study in adolescents**

Monika Frysz, Jennifer S. Gregory, Richard M. Aspden, Lavinia Paternoster, Jonathan H. Tobias

### Supplementary tables and figures

Supplementary Table 1 Variation described by the top ten modes based on adult reference SSM. Abbreviations: FN (femoral neck), NSA (neck-shaft angle)

| HSM  (% of variation) | Key features:  +2 SDs (solid line) -2 SDs (dashed line) | Graphical representation |
| --- | --- | --- |
| 1  (42%) | Positive scores (solid line)   - Loss of femoral head curvature - Narrower FN   Negative scores (dashed line)   - Wider FN Smaller NSA |  |
| 2  (13%) | Positive scores (solid line)   - Narrower FN and shaft - Smaller greater trochanter - Smaller femoral head (inferior aspect proximal to lesser trochanter)   Negative scores (dashed line)   - Wider FN - Larger greater and lesser trochanter - Smaller NSA |  |
| 3 (8.5%) | Positive scores (solid line)   - Smaller femoral head (medial aspect of femoral head) - Narrower femoral neck   Negative scores (dashed line)   - Wider femoral neck - Cam-type deformity |  |
| 4 (6.1%) | Positive scores (solid line)   - Narrower femoral neck - Smaller neck-shaft angle   Negative scores (dashed line)   - Cam type deformity - Wider femoral neck |  |
| 5 (4.1%) | Positive scores (solid line)   - Larger femoral head (inferior aspect proximal to lesser trochanter) - Larger greater trochanter - Wider femoral neck   Negative scores (dashed line)   - Smaller femoral head (inferior aspect proximal to lesser trochanter) - Narrower femoral neck |  |
| 6 (3.4%) | Positive scores (solid line)   - Narrower femoral neck - Smaller femoral head   Negative scores (dashed line)   - Larger femoral head - Wider femoral neck - Decreased acetabular coverage |  |
| 7 (2.6%) | Positive scores (solid line)   - Larger femoral head (medial aspect and inferior aspect proximal to lesser trochanter) - Increased acetabular coverage   Negative scores (dashed line)   - Smaller femoral head (medial aspect and inferior aspect proximal to lesser trochanter) |  |
| 8 (2.5%) | Positive scores (solid line)   - Larger femoral head - Narrower femoral neck   Negative scores (dashed line)   - Smaller femoral head - Larger greater trochanter - Loss of femoral neck to head curvature |  |
| 9 (1.8%) | Positive scores (solid line)   - Smaller lesser trochanter   Negative scores (dashed line)   - Larger femoral head (inferior aspect proximal to lesser trochanter) |  |
| 10 (1.5%) | Positive scores (solid line)   - Larger lesser trochanter   Negative scores (dashed line)   - Smaller lesser trochanter |  |

Supplementary Table 2 Summary statistics for aPHV and tempo in males and females

|  | **Tempo percentile** | **Mean aPHV (min; max)** | **Mean tempo (min; max)** | **N** |
| --- | --- | --- | --- | --- |
| **Males N=1,797** | 10 | 12.1 (10.8; 12.9) | -1.41 (-2.68; -1.05) | 180 |
|  | 20 | 12.6 (12.3; 13.0) | -0.85 (-1.04; -0.68) | 180 |
|  | 30 | 12.9 (12.6; 13.4) | -0.55 (-0.68; -0.42) | 180 |
|  | 40 | 13.2 (13.0; 13.6) | -0.31 (-0.42; -0.21) | 179 |
|  | 50 | 13.4 (13.1; 13.7) | -0.09 (-0.21; 0.04) | 180 |
|  | 60 | 13.7 (13.4; 13.9) | 0.14 (0.04; 0.25) | 180 |
|  | 70 | 13.9 (13.6; 14.1) | 0.35 (0.25; 0.46) | 179 |
|  | 80 | 14.1 (13.8; 14.4) | 0.59 (0.46; 0.70) | 180 |
|  | 90 | 14.4 (14.2; 14.7) | 0.83 (0.70; 1.01) | 180 |
|  | 100 | 15.1 (14.5; 16.6) | 1.47 (1.01; 2.66) | 179 |
|  | **< 90** | 13.2 (10.8; 14.4) | -0.27 (-2.7; 0.70) | 1,438 |
|  | **⩾90** | 14.8 (14.2; 16.6) | 1.15 (0.70; 2.7) | 359 |
|  |  |  |  |  |
| **Females N=2,030** | 10 | 10.5 (9.1 - 10.8) | -1.41 (-2.68; -1.05) | 203 |
|  | 20 | 11.0 (10.6 - 11.2) | -0.85 (-1.05; -0.69) | 203 |
|  | 30 | 11.3 (11.0 - 11.5) | -0.56 (-0.69; -0.44) | 203 |
|  | 40 | 11.5 (11.3 - 11.7) | -0.32 (-0.44; -0.21) | 203 |
|  | 50 | 11.7 (11.5 - 11.9) | -0.09 (-0.21; 0.02) | 203 |
|  | 60 | 11.9 (11.7 - 12.1) | 0.14 (0.02; 0.25) | 203 |
|  | 70 | 12.1 (11.9 - 12.4) | 0.36 (0.25; 0.49) | 203 |
|  | 80 | 12.4 (12.1 - 12.6) | 0.62 (0.49; 0.76) | 203 |
|  | 90 | 12.7 (12.4 - 13.1) | 0.94 (0.76; 1.19) | 203 |
|  | 100 | 13.3 (12.9 - 14.5) | 1.60 (1.20; 3.00) | 203 |
|  | **< 90** | 11.6 (9.1; 12.6) | -0.26 (-2.7; 0.76) | 1,624 |
|  | **⩾90** | 13.0 (12.4; 14.5) | 1.27 (0.76; 3.0) | 406 |

Abbreviations: aPHV (age at peak height velocity)

Supplementary Table 3 Differences in hip shape mode scores at age 14 between those in 90^th^ tempo percentile and above vs. those below 90^th^ tempo percentile, stratified by sex (betas were scaled to represent per-year difference in tempo)

|  |  | **Model 1** | | **Model 2** | |
| --- | --- | --- | --- | --- | --- |
| **Males** | **HSM** | **β (95% CI)** | **p-value** | **β (95% CI)** | **p-value** |
|  | 1 | -0.07 (-0.11, -0.04) | 3.89x10^-5^ | -0.08 (-0.11, -0.04) | 2.61x10^-5^ |
|  | 2 | 0.28 (0.21, 0.34) | 1.7x10^-18^ | 0.29 (0.23, 0.35) | 2.76x10^-21^ |
|  | 3 | 0.25 (0.20, 0.31) | 6.53 x10^-19^ | 0.27 (0.21, 0.32) | 4.29x10^-21^ |
|  | 4 | 0.15 (0.09, 0.21) | 1.72 x10^-7^ | 0.15 (0.09, 0.20) | 2.09x10^-7^ |
|  | 5 | 0.33 (0.27, 0.40) | 1.72 x10^-24^ | 0.33 (0.27, 0.40) | 1.86x10^-24^ |
|  | 6 | 0.01 (-0.04, 0.07) | 0.627 | 0.03 (-0.03, 0.09) | 0.367 |
|  | 7 | -0.21 (-0.26, -0.16) | 8.76 x10^-17^ | -0.20 (-0.25, -0.15) | 1.1x10^-15^ |
|  | 8 | 0.45 (0.38, 0.52) | 1.32 x10^-32^ | 0.46 (0.39, 0.53) | 4.17x10^-34^ |
|  | 9 | 0.19 (0.13, 0.25) | 7.88 x10^-10^ | 0.19 (0.13, 0.25) | 6.63x10^-10^ |
|  | 10 | 0.03 (-0.01, 0.08) | 0.162 | 0.05 (-0.00, 0.09) | 0.067 |
|  |  |  | |  | |
| **Females** | 1 | -0.02 (-0.04, 0.01) | 0.252 | -0.01 (-0.04, 0.01) | 0.353 |
|  | 2 | 0.09 (0.04, 0.15) | 0.001 | 0.04 (-0.01, 0.09) | 0.137 |
|  | 3 | 0.09 (0.04, 0.14) | 2.09x10^-4^ | 0.05 (0.00, 0.10) | 0.037 |
|  | 4 | -0.02 (-0.06, 0.03) | 0.438 | -0.03 (-0.08, 0.02) | 0.243 |
|  | 5 | 0.10 (0.04, 0.15) | 6.51x10^-4^ | 0.12 (0.06, 0.17) | 3.15x10^-5^ |
|  | 6 | -0.08 (-0.12, -0.03) | 0.001 | -0.10 (-0.14, -0.05) | 1.69x10^-5^ |
|  | 7 | 0.04 (-0.01, 0.08) | 0.089 | 0.03 (-0.02, 0.07) | 0.268 |
|  | 8 | 0.18 (0.12, 0.24) | 3.81x10^-9^ | 0.15 (0.09, 0.21) | 2.02x10^-6^ |
|  | 9 | 0.08 (0.02, 0.13) | 0.006 | 0.08 (0.03, 0.14) | 0.003 |
|  | 10 | 0.06 (0.02, 0.11) | 0.002 | 0.04 (0.00, 0.08) | 0.042 |

Abbreviations: HSM (hip shape mode), CI (confidence interval). Table shows results of linear regression analysis showing the difference the top ten HSM scores between two tempo percentile groups in males (those in the 90^th^ and above (N=359) vs those below 90^th^ percentile of tempo (N=1,438)) and females (those in the 90^th^ and above (N=406) vs those below 90^th^ percentile of tempo (N=1,624)). Results are SD difference in HSM per-year increase in tempo in late maturers vs the rest, 95% CIs and p value. Model 1: unadjusted; model 2: adjusted for fat mass index.

Supplementary Table 4 Differences in hip shape mode scores at age 14 between those in 10^th^ tempo percentile vs. those above 10^th^ tempo percentile, stratified by sex (betas were scaled to represent per-year difference in tempo)

|  |  | **Model 1** | | **Model 2** | |
| --- | --- | --- | --- | --- | --- |
| Males | **HSM** | **β (95% CI)** | **p-value** | **β (95% CI)** | **p-value** |
|  | 1 | 0.08 (0.03,0.11) | 0.001 | 0.07 (0.03,0.11) | 8.5 x10^-4^ |
|  | 2 | -0.16 (-0.23,-0.09) | 2.4 x10^-5^ | -0.14 (-0.21,-0.07) | 1.3 x10^-4^ |
|  | 3 | -0.24 (-0.31,-0.18) | 2.0 x10^-13^ | -0.24 (-0.30,-0.17) | 1.8 x10^-12^ |
|  | 4 | 0.01 (-0.06,0.08) | 0.853 | 0.01 (-0.06,0.07) | 0.886 |
|  | 5 | -0.23 (-0.30,-0.14) | 1.0 x10^-8^ | -0.23 (-0.30,-0.15) | 7.8 x10^-9^ |
|  | 6 | 0.10 (0.03,0.18) | 0.004 | 0.12 (0.04,0.19) | 0.001 |
|  | 7 | 0.09 (0.04,0.16) | 0.001 | 0.11 (0.05,0.16) | 3.8 x10^-4^ |
|  | 8 | -0.34 (-0.43,-0.25) | 1.3 x10^-13^ | -0.33 (-0.42,-0.24) | 4.2 x10^-13^ |
|  | 9 | -0.19 (-0.26,-0.12) | 2.8 x10^-7^ | -0.19 (-0.26,-0.12) | 2.8 x10^-7^ |
|  | 10 | -0.11 (-0.16,-0.05) | 3.8 x10^-4^ | -0.09 (-0.15,-0.04) | 0.001 |
|  |  |  |  |  |  |
| Females | 1 | 0.00 (-0.04,0.03) | 0.918 | -0.01 (-0.05,0.03) | 0.720 |
|  | 2 | -0.11 (-0.18,-0.03) | 0.004 | -0.03 (-0.10,0.04) | 0.415 |
|  | 3 | -0.02 (-0.09,0.04) | 0.521 | 0.04 (-0.03,0.10) | 0.232 |
|  | 4 | -0.03 (-0.09,0.03) | 0.392 | -0.01 (-0.08,0.05) | 0.634 |
|  | 5 | 0.09 (0.01,0.17) | 0.018 | 0.07 (-0.01,0.14) | 0.087 |
|  | 6 | 0.01 (-0.06,0.07) | 0.910 | 0.03 (-0.03,0.09) | 0.311 |
|  | 7 | -0.08 (-0.14,-0.02) | 0.009 | -0.06 (-0.13,0.00) | 0.045 |
|  | 8 | -0.05 (-0.13,0.04) | 0.293 | 0.01 (-0.07,0.09) | 0.769 |
|  | 9 | -0.01 (-0.08,0.07) | 0.847 | -0.01 (-0.09,0.06) | 0.734 |
|  | 10 | 0.01 (-0.05,0.07) | 0.776 | 0.04 (-0.01,0.10) | 0.139 |

Abbreviations: HSM (hip shape mode), CI (confidence interval). Table shows results of linear regression analysis showing the difference the top ten HSM scores between two tempo percentile groups in males (those in the 10^th^ (N=180) vs those above 10^th^ percentile of tempo (N=1,617)) and females (those in the 10^th^ (N=203) vs those above 10^th^ percentile of tempo (N=1,827)). Results are SD difference in HSM per-year increase in tempo in early maturers vs the rest, 95% CIs and p value. Model 1: unadjusted; model 2: adjusted for fat mass index.


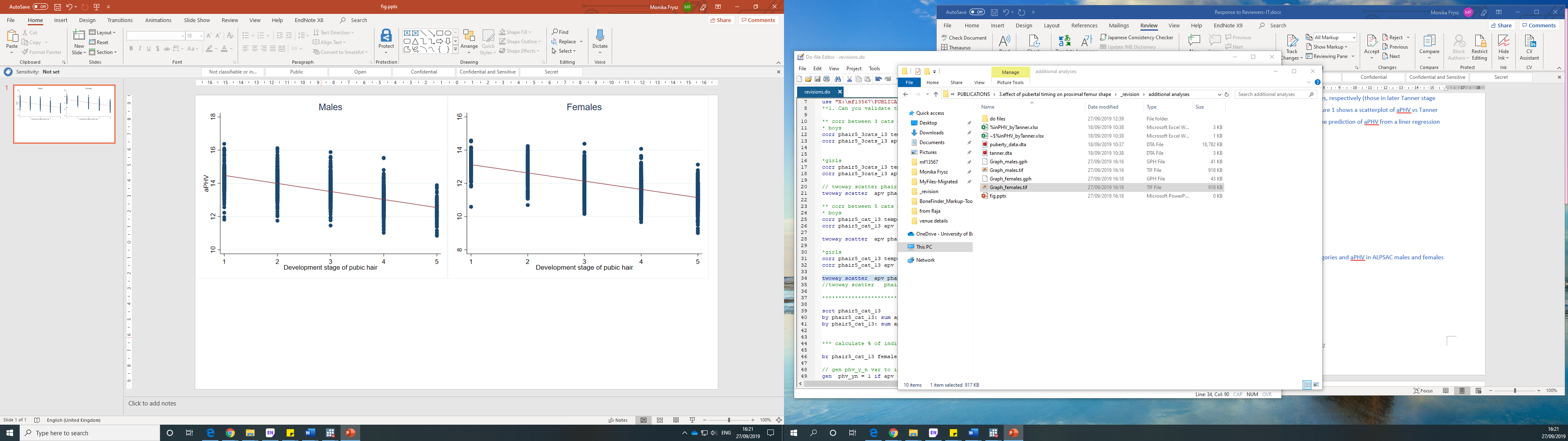


Supplementary Figure 1 Relationship between Tanner stage categories and aPHV in ALSPAC males and females. Red line represents fitted regression line for the prediction of aPHV from a liner regression of aPHV on Tanner stage. Blue dots are age at peak height velocity per Tanner stage category.
